# Inhibition of poly(ADP-ribose) polymerase-1 attenuates the toxicity of carbon tetrachloride

**DOI:** 10.3109/14756366.2011.557315

**Published:** 2011-03-14

**Authors:** Marek Banasik, Todd Stedeford, Robert P Strosznajder, Masanori Takehashi, Seigo Tanaka, Kunihiro Ueda

**Affiliations:** 1 Institute of Public Health and Environmental Protection, Warsaw, Poland; 2 Department of Environmental and Occupational Health, College of Public Health, University of South Florida, Tampa, FL, USA; 3 Department of Neurosurgery, M. Mossakowski Medical Research Centre, Polish Academy of Sciences, Warsaw, Poland; 4 Laboratory of Molecular Clinical Chemistry, Faculty of Pharmacy, Osaka-Ohtani University, Osaka, Japan; 5 Kobe Tokiwa University, Kobe, Japan

**Keywords:** PARP inhibitors, poly(ADP-ribose) synthetase, hepatoprotection, necrosis, apoptosis

## Abstract

Carbon tetrachloride (CCl_4_) is routinely used as a model compound for eliciting centrilobular hepatotoxicity. It can be bioactivated to the trichloromethyl radical, which causes extensive lipid peroxidation and ultimately cell death by necrosis. Overactivation of poly(ADP-ribose) polymerase-1 (PARP-1) can rapidly reduce the levels of (β-nicotinamide adenine dinucleotide and adenosine triphosphate and ultimately promote necrosis. The aim of this study was to determine whether inhibition of PARP-1 could decrease CCl_4_-induced hepatotoxicity, as measured by degree of poly(ADP-ribosyl)ation, serum levels of lactate dehydrogenase (LDH), lipid peroxidation,and oxidative DNA damage. For this purpose, male ICR mice were administered intraperitoneally a hepatotoxic dose of CCl_4_ with or without 6(5*H*)-phenanthridinone, a potent inhibitor of PARP-1. Animals treated with CCl_4_ exhibited extensive poly(ADP-ribosyl)ation in centrilobular hepatocytes, elevated serum levels of LDH, and increased lipid peroxidation. In contrast, animals treated concomitantly with CCl_4_ and 6(5*H*)-phenanthridinone showed significantly lower levels of poly(ADP-ribosyl) ation, serum LDH, and lipid peroxidation. No changes were observed in the levels of oxidative DNA damage regardless of treatment. These results demonstrated that the hepatotoxicity of CCl_4_is dependent on the overactivation of PARP-1 and that inhibition of this enzyme attenuates the hepatotoxicity of CCl_4_.

## Introduction

Many pathological conditions are induced by chemical exposures, of which an increase in free radical production has been shown to be a primary and ubiquitous event in producing toxicity^1^. Oxidative stress or chemical insults that are capable of generating DNA strand breaks cause a remarkable increase in poly(ADP-ribose) polymerase-1 (PARP-1; EC 2.4.2.30) activity^2^, whose activity is barely detectable under normal cellular conditions. When DNA damage is moderate, PARP-1 works for its repair. Upon binding to DNA strand termini, PARP-1 catalyzes the splitting of the ADP-ribose-nicotinamide bond in β-nicotinamide adenine dinucleotide (NAD^+^) with the concomitant attachment of ADP-ribose moiety to protein and then to this protein-bound ADP-ribose, resulting in a long and branched chain of up to 200 units, i.e. poly(ADP-ribose)^3^,^4^. Various proteins serve as acceptors of ADP-ribose. In addition, PARP-1 catalyzes the transfer of ADP-ribose moieties to acceptor sites on itself, a process called “automodification”^3^,^4^. This automodification leads to the release of PARP-1 from DNA strand termini, due to the negative charge repulsion between poly(ADP-ribose) and DNA, and allows for other proteins of the DNA excision repair pathways to access and work at sites of damage.

Overactivation of PARP-1 potentiates the deleterious effects of an initial chemical insult by rapidly depleting cellular NAD^+^ and adenosine triphosphate (ATP) and leads to an “energy crisis” that culminates in cell death^5^-^10^. In such situations, PARP-1 inhibitors very often protect cells from death^8^,^9^,^11^-^15^. It seems that PARP-1 is important for both, apoptotic and necrotic, modes of cell death, but in different ways^16^-^19^. DNA-damage-induced PARP-1 overactivation leads to ATP depletion and necrotic cell death^10^,^20^,^21^. Consequently, PARP-1 inhibition attenuates or prevents necrosis in various cell types^11^,^12^,^21^-^24^. During classical apoptosis (i.e., caspase-dependent programmed cell death), PARP-1 is cleaved by caspases resulting in its inactivation^25^. PARP-1 cleavage, a hallmark of apoptosis, may prevent its overactivation and, therefore, ATP depletion and necrosis by preserving cellular energy that is required for apoptosis. Thus, intracellular ATP levels can regulate the mode of cell death^26^,^27^. A similar effect would be expected from PARP-1 inhibition. In fact, inhibition of PARP-1 preserves cellular ATP levels and in turn minimizes execution of the necrotic death pathway^28^. Moreover, the maintenance of intracellular ATP levels associated with PARP-1 inhibition shifts cell death from necrosis to apoptosis and from apoptosis to cell survival^29^. PARP-1 inhibitors (3-aminobenzamide and 4-hydroxyquinazoline) prevent hydrogen peroxide H_2_O_2_-induced ATP depletion with reversion of the mode of cell death from necrosis back to apoptosis^30^. 3-Aminobenzamide restores NAD^+^ and ATP levels and decreases both necrosis and apoptosis caused by H_2_O_2_-injury of PC12 cells^31^. PARP-1 also plays an important role in a caspase-independent programmed cell death that is mediated by the apoptosis-inducing factor, i.e. a mitochondrial flavoprotein that is released and translocated to the nucleus in response to death stimuli^32^,^33^. PARP-1 activation is necessary for this translocation. Furthermore, during excessive poly(ADP-ribosyl)ation-dependent cell death, there is a rapid cross-talk between the nucleus and mitochondria^34^,^35^. Some studies have shown that pharmacological inhibition of PARP-1 is capable of attenuating the adverse effects of selected pharmaceuticals^36^.

Several lines of evidence have shown that the toxicity of carbon tetrachloride (CCl_4_; Chemical Abstracts Service Registry Number: 56-23-5) is dependent on an early efflux of calcium (Ca^2+^) from the endoplasmic reticulum, due to intense lipid peroxidation caused by the trichloromethyl radical^37^,^38^. Late preventive effects of nicotinamide and dimethyl sulfoxide (DMSO) against CCl_4_- and bromobenzene-induced hepatotoxicity have already been reported^39^,^42^. These effects were assumed to be the result of the ability of nicotinamide treatment to restore mitochondrial Ca^2+^ transport and the antioxidant effects of DMSO. More recently, we showed that increased ADP-ribosylation of hepatocellular proteins occurred in mice 24 h after the administration of a hepatotoxic dose of CCl_4_^43^. Further, we found that 6(5*H*)-phenanthridinone (Chemical Abstracts Service Registry Number: 1015-89-0), a potent inhibitor of PARP-1 (IC_50_ = 300 nM)^44^, offered a protective affect against CCl_4_-induced hepatotoxicity by decreasing areas of hepatocellular necrosis and serum transaminase levels^45^. Herein, we report additional parameters from these previous studies that further support the protective effects of PARP-1 inhibition in animals poisoned with CCl_4_.

## Materials and methods

### Reagents and animal treatment

All reagents were purchased from Sigma-Aldrich Corp. (St. Louis, MO), unless otherwise indicated. Experimental evaluations were performed on tissues from animals reported on previously^43^,^45^. Briefly, male ICR mice, weighing approximately 25 g at the time of experiment, were purchased from Harlan Sprague Dawley, Inc. (Indianapolis, IN), housed in an air-conditioned vivarium, with a 12-h light–dark cycle, and allowed free access to diet, *2018 - Teklad Global 18% Protein Rodent Diet* (Harlan Teklad, Madison, WI), and drinking water. Animals were acclimated for 7 days, prior to being randomly assigned to the treatment groups (Table 1). Experimental animals were administered intraperitoneally 200 μL of an appropriate solution and sacrificed by decapitation after 24 h. 6(5*H*)-Phenanthridinone was dissolved in DMSO; final DMSO concentration in injection solutions (0.2 mL), except controls, was 5.5%. Therefore, assuming an average mouse blood volume of 6–8 mL per 100 g of body weight^46^, injecting a 25 g mice with 0.2 mL of 5.5% DMSO would result in a final *in vivo* DMSO concentration of between 0.5 and 0.65%. This study was approved by an Institutional Ethics Committee.

**Table 1:** Experimental design for control and treatment groups.

Group	Addition*	Number of Animals†
PBS	None (only PBS)	4
DMSO‡	11 μL#	4
6(5*H*)-Phenanthridinone§	10 mg/kg‖ in 11 μL#	4
CCl_4_	572 mg/kg + 11 μL#	10
CCl_4_ + 6(5*H*)-Phenanthridinone	572mg/kg + 10 mg/kg in 11 μL»	10

* Total intraperitoneal injection volume, with and without addition(s), was 200 μL as made up with PBS for each group.

† Male ICR mice were preconditioned as described in “Materials and methods” section.

‡ Final concentration of DMSO, when added, in injections was 5.5%, resulting in an *in vivo* concentration of less than 0.65%.

§ 6(5*H*)-Phenanthridinone was dissolved in 100% DMSO.

‖ Kilogram of body weight

# DMSO.

### Measurements of serum enzymes

Mice were decapitated, and whole blood was collected in sterile microcentrifuge tubes, left at room temperature for 5 min, and centrifuged at 5000g for 10 min. The serum (supernatant; ∼0.2 mL) was transferred to a new sterile microcentrifuge tube and stored at 4°C until assay. The pellets were disposed. The activity of lactate dehydrogenase (LDH; EC 1.1.1.27) was determined using a commercial kit.

### Immunohistochemistry and evaluation of poly(ADP-ribosyl)ation

Liver samples previously fixed in 10% neutral buffered formalin and embedded in paraffin were deparaffinized using the xylene/ethanol procedure. After endogenous peroxidase was blocked with 3% H_2_O_2_ in methanol at room temperature for 10 min, the sections were treated with 90, 80, and 70% ethanol for 3 min each. Then, they were washed three times for 3 min with phosphate-buffered saline (PBS). Antigens were unmasked by incubating samples with 0.1% trypsin at 37°C for 30 min, followed by rinsing three times for 3 min with Tris-buffered saline and once for 3 min with containing 0.1% Tween-20 (TBS-T). The sections were reacted with rabbit anti-poly(ADP-ribose) polyclonal antibodies (produced in our laboratory)^47^ diluted 100-fold in TBS-T with 1% bovine serum albumin at 4°C overnight. As a control, parallel tissue sections of PBS- and CCl_4_-treated animals were incubated overnight in TBS-T with 1% bovine serum albumin and no antibody. After washing with TBS-T (3 min × 5), the sections were incubated for 1 h at room temperature with EnVision^+^™ (Code No. K4002; DakoCytomation Denmark A/S, Glostrup, Denmark) and visualized using 3,3'-diaminobenzidine (0.2 mg/mL in PBS containing 0.006% H_2_O_2_).

### Measurement of lipid peroxidation

Hepatic levels of malondialdehyde were measured as a marker of lipid peroxidation, as described previously^43^. Briefly, liver tissues, collected at the end of experiments, were homogenized in 1.15% potassium chloride solution. An aliquot (0.1 mL) of the homogenate was added to a reaction mixture containing 0.2 mL of 8.1% (w/v) sodium dodecyl sulfate, 1.5 mL of 20% (v/v) acetic acid (pH 3.5), 1.5 mL of 0.8% (w/v) thiobarbituric acid, and 0.7 mL distilled water. Samples were then heated for 1 h at 95°C and centrifuged at 3000g for 10 min. The absorbance of the supernatant was measured by spectrophotometry at 650 nm, and the results were reported as thiobarbituric acid reactive substances (TBARS) per milligram of protein.

### Measurement of oxidative DNA damage

DNA isolation, hydrolysis, and analysis of 8-hydroxy-2'-deoxyguanosine (oxo^8^dG), expressed as a ratio of oxo^8^dG to 2-deoxyguanosine (2-dG), was performed as described previously^48^. Briefly, samples were treated with DNase-free RNase followed by digestion with proteinase K. The protein fraction was separated from DNA by three consecutive organic extractions. The DNA was precipitated by adding two volumes of ethanol (with respect to the aqueous volume) and incubated overnight at −20°C. The purity of the DNA was determined by the absorbance of an aliquot of the sample at 260 versus 280 nm.

Analysis of the ratio of oxo^8^dG/2-dG was performed as described previously^48^. Purified DNA was prepared for high-performance liquid chromatography analysis by resolving it into deoxynucleoside components. The DNA was digested with nuclease P1 and treated further with alkaline phosphatase. The deoxynucleosides preparation was then ready for HPLC analysis. The amount of oxo^8^dG and 2-dG was calculated by comparing the peak area of oxo^8^dG and 2-dG obtained from the enzymatic hydrolysate of the DNA sample to a calibration curve for both compounds. Levels of oxo^8^dG in the samples were expressed relative to the content of 2-dG, for example, as the ratio of fmol of oxo^8^dG/nmol of 2-dG. Because 1 μg of DNA contains 0.648 nmol of 2-dG, 1 fmol/nmol 2-dG is equivalent to 1.54 fmol/ μg DNA. The mobile phase in the HPLC system was 100 mM sodium acetate, pH 5.2, with 5% methanol. Oxo^8^dG was detected by an electrochemical detector (ESA Coulochem Model 5100A; ESA Biosciences, Inc., Chelmsford, MA) using a glassy carbon working electrode at an applied potential of +0.4 V. 2-dG was detected in the same sample by absorbance at 260 nm using a Perkin–Elmer 785A Programmable Absorbance Detector (Perkin Elmer Corp., Norwalk, CT) arranged in series with the electrochemical detector. Data were recorded, stored, and analyzed on a PC Pentium computer using ESA 500 Chromatography Data System Software (ESA Biosciences, Inc., Chelmsford, MA).

### Statistical analysis

One-way analysis of variance was used to compare differences between treatment groups for the serum biochemistry, lipid peroxidation, and oxidative DNA damage experiments, followed by a Student–Newman–Keuls post-test. A probability value of less than 5% was considered significant. Statistical tests were performed using GraphPad Prism™ version 3.0 for Windows (GraphPad Software, Inc., San Diego, CA).

## Results

### Protective effects of PARP-1 inhibitor against CCl_4_-induced damage of hepatocytes

**Figure 1:**
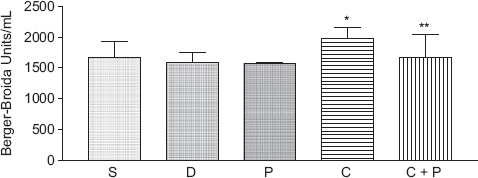
Serum LDH activity for control (S, D, P) and treated (C, C + P) male ICR mice 24 h after treatment. *n* = 4 for PBS (S), DMSO (D), and 6(5*H*)-phenanthridinone (P); *n* =10 for CCl_4_ (C) and CCl_4_ plus 6(5*H*)-phenanthridinone (C + P). Results are expressed as the mean ± the standard deviation. *Indicates a statistically significant difference from controls (*P*<0.05). **Indicates a statistically significant difference between CCl_4_ and CCl_4_ plus 6(5*H*)-phenanthridinone treated animals (*P*<0.05).

Serum LDH activity confirmed the protective effects of 6(5*H*)-phenanthridinone against CCl_4_-induced tissue damage and the lack of effect of DMSO and 6(5*H*)-phenanthridinone administered alone (Figure 1). A statistically significant increase in LDH activity was observed in animals treated with CCl_4_ versus PBS, DMSO, and 6(5*H*)-phenanthridinone controls. A statistically significant decrease in LDH activity was detected between CCl_4_-treated animals and animals treated concomitantly with CCl_4_ and 6(5*H*)-phenanthridinone. No statistically significant difference in LDH activity was detected between animals treated with PBS, DMSO, or 6(5*H*)-phenanthridinone, and animals treated concomitantly with CCl_4_ and 6(5*H*)-phenanthridinone.

### Induction of poly(ADP-ribosyl)ation in hepatocytes by CCl_4_

**Figure 2:**
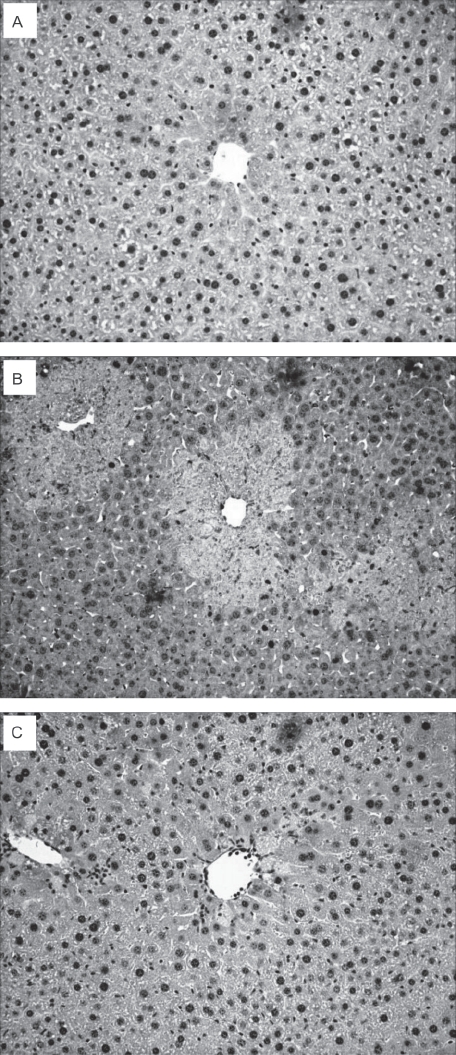
Immunohistochemistry for poly(ADP-ribose) of male ICR mice treated for 24h with CCl_4_. Representative photomicrograph (100×) of [A] PBS controls (S); [B] CCl_4_ (C), and [C] animals co-treated with CCl_4_ and 6(5*H*)-phenanthridinone (C + P). (See colour version of this figure online at http://www.informahealthcare.com/enz)

A minimal degree of poly(ADP-ribosyl)ation of cells was observed in tissues from animals administered PBS (Figure 2A), DMSO (data not shown), or 6(5*H*)-phenanthridinone (data not shown) controls; however, extensive poly(ADP-ribosyl)ation was observed throughout the centrilobular region of CCl_4_-treated animals (Figure 2B). In contrast, animals treated concomitantly with CCl_4_ and 6(5*H*)-phenanthridinone had markedly decreased levels of poly(ADP-ribose) (Figure 2C).

### Induction of lipid peroxidation in hepatocytes by CCl_4_

**Figure 3:**
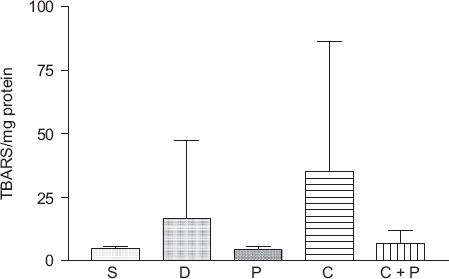
Lipid peroxidation in liver samples of male ICR mice treated with CCl_4_. No statistically significant differences were detected with a *P*<0.05; *n* = 4 for PBS (S), DMSO (D), and 6(5*H*)-phenanthridinone (P); *n* = 9-10 for CCl_4_ (C) and CCl_4_ plus 6(5*H*)-phenanthridinone (C + P).

No statistically significant differences were observed between any groups with regard to markers of lipid peroxidation (Figure 3). However, a 7-fold increase of the average in lipid peroxidation was observed in animals treated with CCl_4_ versus animals treated with either PBS or 6(5*H*)-phenanthridinone. Animals treated concomitantly with CCl_4_ and 6(5*H*)-phenanthridinone exhibited a 5-fold decrease in lipid peroxidation products versus CCl_4_-treated animals. Interestingly, animals treated with DMSO had an approximately 2.5-fold increase in lipid peroxidation products over animals treated concomitantly with CCl_4_ and 6(5*H*)-phenanthridinone and nearly a 3.5-fold increase over PBS and 6(5*H*)-phenanthridinone controls.

### No oxidative DNA damage by CCl_4_

**Figure 4:**
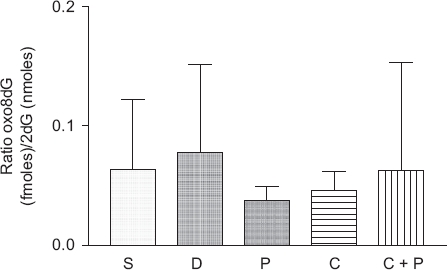
Oxidative DNA damage in liver samples of male ICR mice treated with CCl_4_. No statistically significant differences were detected with a *P*<0.05; *n* = 4 for PBS (S), DMSO (D), and 6(5*H*)-phenanthridinone (P); *n* = 9-10 for CCl_4_ (C) and CCl_4_ plus 6(5*H*)-phenanthridinone (C + P).

The levels of oxidative DNA damage were not statistically significant between any groups (Figure 4). Moreover, no apparent trend appeared to exist with any of the treatments.

## Discussion

This study was undertaken to further evaluate the protective effect of PARP-1 inhibition on CCl_4_-induced hepatotoxicity^49^. The ability of DMSO to intervene in the development of bromobenzene- and chloroform-induced hepatotoxicity has been shown when animals are treated up to 24 h after administration of the toxicant. This effect would be independent of the bioactivation of CCl_4_ to the trichloromethyl radical, which occurs between 6 and 10 h after treatment^49^. The *in vivo* concentration of DMSO utilized in the aforementioned studies was between 2.5 and 3.2% based on the average mouse blood volume of 6–8 mL per 100 g of bodyweight^46^. Interestingly, DMSO has been shown to cause a 20% reduction in PARP-1 activity at a concentration of 4% *in vitro*^50^. Therefore, it was hypothesized that the late protective effects of DMSO may occur, in part, by preventing the overactivation of PARP-1, thereby allowing partially damaged cells to overcome the initial insult through reparative processes or facilitating apoptosis in excessively damaged cells.

Many of the known PARP-1 inhibitors are water insoluble, and organic solvents must be used as the vehicles for administration. DMSO is the most commonly used^50^. Previous studies have shown that DMSO can increase the acute lethality of CCl_4_^51^, but decrease its hepatotoxicity^52^. Furthermore, several studies have shown that in human hepatocytes DMSO can alter the activity of various isoforms of cytochrome P450 (CYP), including CYP2E1 that bioactivates CCl_4_ to the trichloromethyl radical^53^; however, an *in vitro* inhibitory effect was observed only at concentrations greater than 0.1% DMSO. Further, an *in vivo* concentration of DMSO at 1.66% or lower does not provide any protection against bromobenzene- or chloroform-induced hepatotoxicity, both of which are bioactivated by CYP2E1^54^-^56^. As presented herein, the administered amount of DMSO (<0.65% *in vivo)* was maintained sufficiently low enough so as to prevent it from altering the hepatotoxicity of CCl_4_.

An important consideration in interpreting our previous findings and the results presented herein is which of the inhibitory and antioxidant effect of DMSO or the inhibitory effect of 6(5*H*)-phenanthridinone on the bioactivation of CCl_4_ via CYP2E1 dominated. Since 6(5*H*)-phenanthridinone is predominantly eliminated as a glucuronide conjugate and is not further metabolized in benzo[a]pyrene-induced animals^57^,^58^, our results suggest that 6(5*H*)-phenanthridinone protects against CCl_4_-induced hepatotoxicity independently of its metabolism.

Recently, Cover et al.^59^ suggested that PARP-1 activation might not contribute to acetaminophen-induced cell death under the experimental conditions used in their study. In the report, two PARP-1 inhibitors were tested and gave mixed results. 3-Aminobenzamide completely protected against acetaminophen hepatotoxicity, whereas 5-aminoisoquinolinone lacked protective effects. The authors hypothesized that 3-aminobenzamide might reduce metabolic activation of acetaminophen or act as an antioxidant, and, in view of the results with the more potent inhibitor 5-aminoisoquinoline (IC_50_=10 μM), concluded that PARP-1 activation was not a relevant event for acetaminophen-induced oncotic necrosis. Based on their results, it is possible that the 5-aminoisoquinoline used in the study was partially degraded or rapidly metabolized when administered by the intraperitoneal route, as the administered dose did not appear to inhibit PARP-1 to the degree that such a potent inhibitor should inhibit. For instance, the authors report that the staining intensity of poly(ADP-ribose)-positive cells was not significantly reduced in animals treated with acetaminophen and 5-aminoisoquinoline.

Alternatively, the discrepancy between the results of Cover et al.^59^ and the results presented herein may be due to the different pathways by which acetaminophen and CCl_4_ cause hepatotoxicity. For instance, the metabolic activation of acetaminophen generates a reactive metabolite, *N*-acetyl-*p*-benzoquinone imine, that covalently binds to cellular proteins, whereas the bioactivation of CCl_4_ to the trichloromethyl free radical results in lipid peroxidation. Wan et al.^60^ showed that metabolic activation of acetaminophen resulted in an increase in oxidative DNA damage, due, in part, to significantly reduced levels of 8-oxoguanosine DNA glycosylase, a base-excision repair enzyme that removes oxidatively modified DNA bases. In comparison, the types of DNA lesions, commonly called ethenobases, resulting from CCl_4_ are predominantly those originating from products of lipid peroxidation (e.g. malondialdehyde and 4-hydroxynonenol) and not an increase in oxo^8^dG, as shown herein. However, the ethenobases are repaired via the base-excision repair pathway, specifically alkyl-*N*-purine-DNA glycosylase (ANPG). The recent findings of Ogawa et al.^61^ indicate that differences exist with regard to the protective effects of PARP-1 inhibition and the type of DNA damage to be repaired via the nucleotide-excision repair pathway or the base-excision repair pathway, with inhibitors conferring a protective effect in the latter case. It is possible that inactivation of Ogg1 during acetaminophen intoxication prevents the activation of PARP-1, given that glycosylases initiate base-excision repair by removing the altered DNA base, followed by excision of the sugar-phosphate backbone, which triggers the activation of PARP-1. Since CCl_4_ generates lipid peroxidation with the subsequent formation of ethenobases, the protective effect of PARP-1 inhibition on CCl_4_ intoxication may be due to a possible increase in ethenobase removal by ANPG, which, in the absence of PARP-1 inhibition, may lead to PARP-1 overactivation. If the activity of ANPG is inducible as with other glycosylases, the enhanced removal of ethenobases may ultimately lead to overactivation of PARP-1 following excision of the sugar-phosphate backbone and DNA strand break formation^62^. Finally, the degree to which a parent compound and its metabolites may or may not alter the activity of PARP-1 remains an area of investigation that has not been readily explored. In studies where high doses of chemicals are used to treat animals, as performed in this study, saturation of metabolic pathways may end up providing some degree of protection if the parent compound inhibits PARP-1. Some insight into such phenomena was recently reported with several different types of heterocyclic amines, many of which inhibited PARP-1 in their unmetabolized form; however, some compounds caused a significant increase in PARP-1 activity^62^.

In summary, our data demonstrated that 6(5*H*)-phenanthridinone treatment attenuated CCl_4_-induced hepatotoxicity. This effect does not appear to be a result of the use of DMSO as a carrier because the DMSO + CCl_4_ produced intense centrilobular necrosis. Since 6(5*H*)-phenanthridinone is predominantly eliminated as a glucuronide conjugate and is not further metabolized in control or benzo[a]pyrene-induced animals [57, 58], our results suggest that 6(5*H*)-phenanthridinone protects against CCl_4_-induced centrilobular hepatotoxicity without altering CCl_4_ bioactivation. Moreover, when viewed in toto with our previous findings, these results show the predominant role of PARP-1 overactivation in chemical-induced hepatotoxicity and strongly suggest the possibility of pharmacological intervention with PARP-1 inhibitors for chemical-induced hepatotoxicity and possibly other cytotoxicities.
